# Awareness Regarding Medicolegal Aspects of Medical Services Among Reproductive Age Women: A Population-Based Cross-Sectional Study

**DOI:** 10.7759/cureus.49360

**Published:** 2023-11-24

**Authors:** Garima Kachhawa, Asmita Kaundal, Vidushi Kulshrestha, Divya Sethi, Alka Kriplani, V Sreeniwas, Nutan Agarwal

**Affiliations:** 1 Obstetrics and Gynaecology, All India Institute of Medical Sciences, New Delhi, IND; 2 Obstetrics and Gynaecology, AIl India Institute of Medical Sciences, Bilaspur, IND; 3 Obstetrics and Gynaecology, AIl India Institute of Medical Sciences, New Delhi, IND; 4 Biostatistics, All India Institute of Medical Sciences, New Delhi, IND; 5 Obstetrics and Gynecology, Paras Hospitals, Gurgaon, IND

**Keywords:** consumer protection act, mtp act, pcpndt act, medicolegal, awareness

## Abstract

Introduction: The government has implemented various laws to regulate medical practice and improve the quality of health care services. This study evaluated the general population's awareness of various medicolegal aspects related to the medical profession. Methods: A cross-sectional study was conducted. Knowledge of laws and ethics related to medical practice was assessed based on a well-structured questionnaire including 25 items. Women were categorized based on their score into low (below 50th percentile), medium (50th -75th percentile), and high (above 75th percentile) awareness. Results: A total of 334 women were recruited. The mean age of the women in the study was 30.29±6.58 SD years; most women were between 20-30 (56.28%). Most women were graduates (33.23%), followed by postgraduates (29.04%). The majority of women were unemployed (housewives: 64.67%, students: 4.49%), followed by skilled workers (22.75%), semi-professional, and professionals (8.08%). High awareness about the various medicolegal aspects was seen in 25.1% of women, while 29.04% had medium awareness and 45.80% had low awareness. It was also seen that the women with higher education(p=0.002) and those employed (0.001) had better knowledge. Further, graduate housewives had better awareness than non-graduate housewives. Conclusion: Education and self-independence significantly affected awareness of medicolegal issues among our women. Assuring the right to education and empowering women with self-independence will go a long way in ensuring active participation in medical decision-making.

## Introduction

Due to considerable changes in the medical practice in the past few years, it was realized that the medical profession needs to be regularized, and hence, various laws have been implemented [1]. These laws aim to involve the patient in every aspect of their treatment, prevent malpractice by medical professionals, and improve the quality of healthcare services at all possible levels [2-4]. Medical Termination Act (1971) and Preconception Prenatal Diagnostic Test Act (1994) were launched to prevent in-utero sex determination, female feticide, and unsafe abortions [5-7]. Protection of Children from sexual offenses Act (2012) was introduced to deal with sexual offenses against children under 18 years of age. To safeguard patients from medical negligence and to enable them to file lawsuits, the medical profession was covered under the Consumer Protection Act (1995) [8-12]. Despite various laws and efforts by the government, the quality of healthcare facilities remains poor. For a successful implementation of any law, there must be adequate knowledge and awareness about the law among providers and the recipient. Inadequate knowledge, lack of awareness, and inappropriate attitudes towards health services are reasons for the failure of quality care in health services.

Hence, this study was done to evaluate awareness about the medicolegal aspects of medical service among women in the reproductive age group who attended the Gynaecology OPD for various health issues and to correlate the awareness level with the education and occupation of the participants. 

## Materials and methods

This cross-sectional study was done in the Department of Obstetrics and Gynaecology, All India Institute of Medical Sciences, New Delhi, India. Women between the ages of 18 and 45 who understand and can read and write either English or Hindi were included in the study. Women who were already aware of the subject, like lawyers, doctors, nurses, counselors, NGO workers, or their family members who filed a legal suit or complaint about medical negligence, were excluded from the study. Informed written consent was taken after the women read the information sheet and understood the plan and purpose of the study. A self-administered, well-structured questionnaire including 25 items was prepared both in English and Hindi to evaluate the awareness and practice regarding the Consumer Protection Act (items 1-4), Awareness regarding confidentiality, consent, and general medicolegal aspects (items 5-13 and 23-25), Preconception and prenatal diagnostic test (PCPNDT) (item 14-17), and Medical termination of pregnancy (MTP) act (item 18-22) (Table 3). Before data collection, the questions were pretested among 20 women to ensure validity and degree of repeatability. The participants were then asked to respond to each item according to the response format provided in the questionnaire, which the examiner later checked. Each correct response was given one point, and the wrong or unanswered question was given zero points. Total marks were added, and the median was calculated based on which they were categorized into three categories. Those who scored less than the 50th percentile were categorized as Category 1 (Low awareness), those between the 50th -and 75th percentile were categorized as Category 2 (Medium awareness), and those above the 75th percentile were Category 3 (High awareness). 

Study design: Population-based cross-sectional study

The data was analyzed using SPSS version 23.00 software. Descriptive statistics were obtained, and frequency distribution, means, and standard deviation were calculated for awareness among patients regarding various medicolegal issues. The Chi-square test has been used to find the significance of the study parameters on a categorical scale between two or more groups. Non - parametric settings used for qualitative data analysis. Fisher exact test was used when the cell sample was very small.

## Results

**Figure 1:**
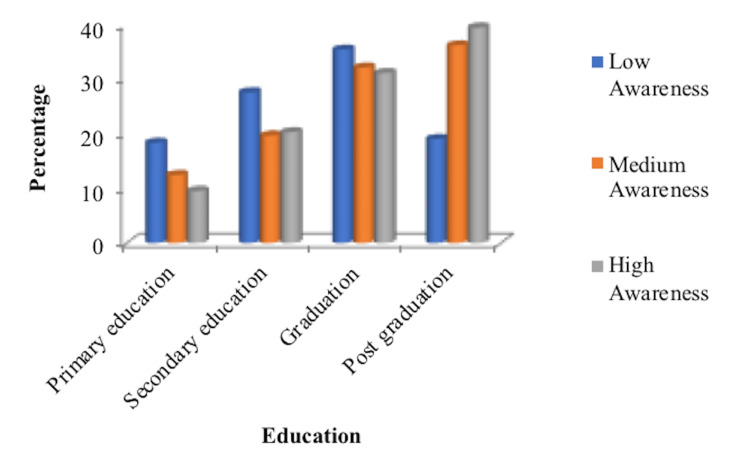
Awareness level with respect to the education status of the participants

A total of 450 women were briefed about the study, out of which 108 refused due to lack of time and interest. Only 334 women filled out the questionnaire and were available for analysis. The mean age of the women in the study was 30.29±6.58SD years. Most participants were graduates (33.23%) and postgraduates (29.04%). The majority were housewives (64.67%) (Table 1). Most participants exhibited low awareness of 45.80% for medicolegal aspects of health services. However, 29.04% had medium awareness, and 25.14% had high awareness. When the results were compared with the education status of the women, it was found that women with higher education were more aware than those with lower education levels, and the difference was statistically significant (P = 0.015) (Figure 1).

**Table 1:** Demographic profile of the study participants

Age (Years)	Percentage (%)
<20 (n=7)	2.09
20-30 (n=188)	56.28
30-40 (n=110)	32.93
>40 (n=29)	8.68
Education	
Primary (n=48)	14.37
Secondary (n=78)	23.35
Graduate (n=111)	33.23
Postgraduate and above (n=97)	29.04
Occupation	
Housewives (n=216)	64.67
Skilled (n=76)	22.75
Semi-professional(n=16)	4.79
Professional (n=11)	3.29
Students (n=15)	4.49

**Figure 2:**
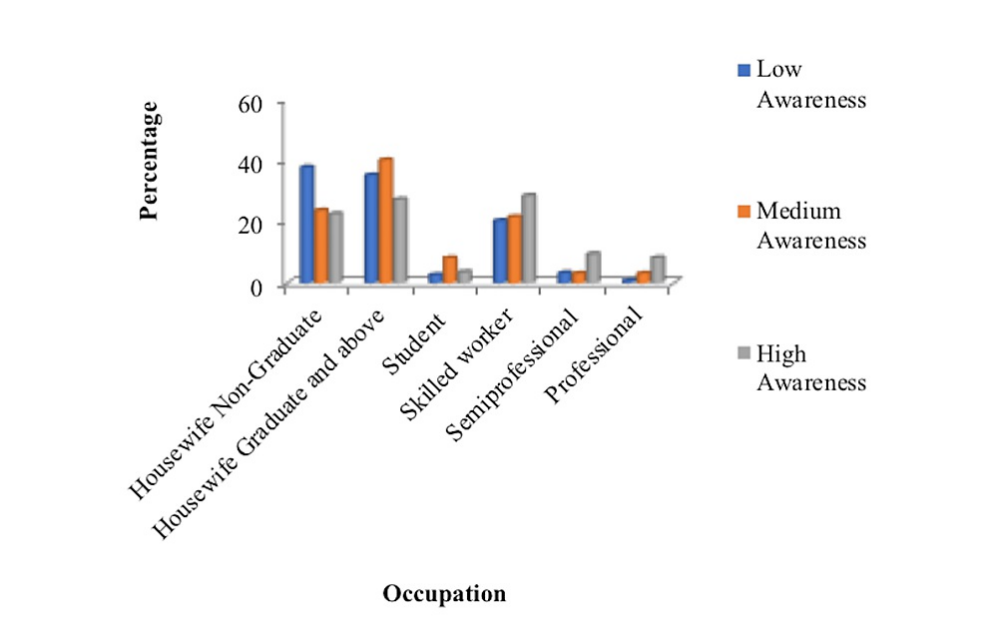
Awareness in relation to the occupation of the participants

Similarly, the results were related to the occupation of the women and their awareness of the medicolegal issues. A statistically significant difference was seen in the knowledge of women who were employed compared to those who were not working (p=0.001) (Figure 2). Even in the subset of women who were graduates and above, it was seen that women working had better knowledge than those who were unemployed (p-0.002). The data were further evaluated to see the awareness about the PCPNDT and MTP act, and it was seen that the awareness of those highly educated and employed was more than that of those with lower education and unemployment (Table 2).

**Table 2:** Awareness in relation to the education level and occupation of the study participants

Variable	Low awareness (n=153)	Medium awareness (n=97)	High awareness (n=84)	P-Value
Wife Education				
Primary (n=48)	28 (18.3%)	12 (12.4%)	8 (9.5%)	0.015
Secondary (n=78)	42 (27.5%)	19 (19.6%)	17 (20.2%)	
Graduate (n=111)	54 (35.3%)	31 (32.0%)	26 (31.0%)	
Postgraduate (n=97)	29 (19.0%)	35 (36.1%)	33 (39.3%)	
Wife Occupation				
Student (n=15)	4 (2.6%)	8 (8.2%)	3 (3.6%)	0.001
Skilled (n=76)	31 (20.3%)	21 (21.6%)	24 (28.6%)	
Semi-professional (n=16)	5 (3.3%)	3 (3.1%)	8 (9.5%)	
Professional (n=11)	1 (0.7%)	3 (3.1%)	7 (9.5%)	
Housewife Graduate (n=116)	54 (35.3%)	39 (40.2%)	23 (27.4%)	
Housewife Non-graduate (n=100)	58 (37.9%)	23 (23.7%)	19 (22.6%)	

## Discussion

The government has implemented several laws to ensure the safety and quality of patient care, but the knowledge of these laws and medical ethics remains poor among the general population. Ignorance, illiteracy, and neglected medicolegal services are the major contributors to these failures. 

Very few studies are done to examine patients' and physicians' knowledge of the medicolegal aspects of healthcare facilities. In a study done in Malaysia by Yousuf et al., 85% of patients were reasonably informed about their illness [13]. Similar findings were seen in the present study, where 81.7% of women wanted to know about their diagnosis whenever they visited a doctor. However, 18.3% did not bother to know what was wrong with them but were satisfied by just getting treated. Although the doctor must explain to the patient the problem he or she is suffering from, it is equally important for the patient to enquire about his or her health issues. Only then will they be able to care for themselves and ensure follow-up. In India, where various forms of medicine are practiced and various levels of health care providers range from general physicians to super-specialists, the patient must know about their health service providers' specialty and expertise. Around 60.8% of women knew about treating doctors' qualifications, while 39.2% were unaware. This implies that these women are unaware of whom to consult for a particular problem and hence lose a significant amount of their valuable time and money consulting one after another doctor. If the patient is aware of the doctor's field of expertise, they might make a better choice while choosing their treating doctor based upon the doctor's qualification, hence decreasing unnecessary referrals. In this study, 38.1% of women were not aware that doctors also come under the Consumer Protection Act, which means such a large population does not know where to go if they need to complain about malpractice and negligence if the need arises.

In India, the Medical Termination of Pregnancy (MTP) Act was implemented in 1971 and has been recently amended in 2021. The act was implemented with the intention of reducing illegal abortions and maternal mortality due to unsafe abortion practices. Awareness about the act among the general population is low [14]. Around 45.47% of women in the present study did not know that the doctors performing MTP should be certified. Only 29.9% of the women were aware of the legal limit of gestational age till which abortion can be done, leading to delays in seeking medical help, resulting in a denial of abortion services at advanced gestation, which further increases the risk of illegal abortions, increasing maternal morbidity and mortality. This also increases the number of abandoned newborns born as a result of unwanted pregnancies among unmarried women. Around 13.2% of the women did not know that their medical records and information are kept confidential which can be a significant contributor to not seeking medical advice or delay in seeking medical advice in certain situations like unwanted pregnancies among adolescent girls and going for unsupervised abortions further increasing the burden of maternal mortality [15]. 

While assessing the awareness about the PCPNDT Act, it was seen that only 47.6% of women were aware of the PCPNDT Act. Around 75.4% of women felt it is inappropriate to do the sex determination, while 11.7% felt it's okay, and 12.9 % were not sure again, leaving around 24.6% of women in the grey zone who could go for prenatal detection for sex willingly or under pressure from the family further increasing their chances of female feticide, illegal abortions at inappropriate gestations by an underqualified person even posing a threat to the life of the mother undergoing an abortion. Similar to our study, Shidhaye PR also found that around 73.5% of women were aware of the availability of methods for intrauterine sex determination, but only 75% knew that it is a crime and is punishable [16]. Another study done by Puri et al. found that 65% of women feel it is not appropriate to determine the sex of the child in utero, but only 16% were aware that it is punishable [17]. So, there are so many areas where intensive work is needed to improve the knowledge about health care quality. The doctor and the general population need to be educated about the services available, how to procure them, and the legal implications for both the patient and the doctor. 

Around 41.9% of the patients in the present study felt a lack of communication between them and the doctor treating them. Similar results were seen in a study by Kumar Manisha et al., where authors found that most doctors (52.3%) spent less time explaining the disease and treatment being provided to the patient [18]. Thus, doctors need to be trained in better communication with the patient to understand the patient's needs and give detailed information regarding the disease, prognosis, treatment options, and advantages and disadvantages of various available treatment modalities. All this communication should be done in simple, understandable language to the patient. If referrals are needed, make sure to explain the need for referral. The authors found that the literacy level was related to the patient's attitude and understanding of the consent.

Similarly, we have also seen that education was directly related to women's awareness of different health services and laws. A study by Vadera et al. has also shown the impact of education on patient awareness [19]. In the present study, we have also seen that the level of awareness in women was directly related to their education level and more to their occupation. Educated, working, and self-independent women are more aware and make better choices. We have seen that unemployed women, even when they are educated, have less knowledge and awareness about various medicolegal aspects than those who are employed, probably because employed women get the opportunity to come out and discuss different issues with other people. Meeting more people and more exposure to the outside world might be a possible reason for their higher knowledge. 

To successfully implement any policy, awareness in the general population must be raised. Television, radio, healthcare-related messages on mobile phones, appropriate mobile apps, free health check-ups, and health talks are available. However, awareness, perception, and acceptance of these must be enhanced. Educational material can be provided to the patients and their attendants in local languages to make them aware of their rights, various acts, and policies. Education via nuked natak and awareness camps are also ways to spread awareness regarding different medical aspects of the medical profession.

## Conclusions

Safe and quality medical care is the fundamental right of every individual. The state implements various laws and acts to provide quality medical care and safeguard the general public's rights. But to maximize the utilization of these quality services, it is important to spread awareness of these laws in the community to enable them to make more informed choices about their medical needs.

## Appendices

**Table 3:** Questionnaire

Question No.	Question	Response
1.	Are you aware of your consulting doctor’s degree/qualification?	a. Yes- 60.8% b. No-34.1% c. Did not understand -5.1%
2.	Do you know that all doctors have to be registered with the Medical Council of India (MCI) before practice?	a. Yes-75.7% b. No-12.9% c. Did not understand -11.4%
3.	Do You know about the Consumer Protection Act (CPA)?	a. Yes, heard about but do not understand -18.0% b. Yes, understand it-59.9% c. Not heard about it-22.2%
4.	Do you think doctors are liable for malpractice under the Consumer Protection Act (CPA)?	a. Yes-56% b. No-15.9% c. Did not understand-28.1%
5.	In case one of your relatives/friends feels there has been malpractice in her case, will you advise her to complain?	a. Yes-78.4% b. No.-12.9% c. Did not understand-8.7%
6.	If the answer to the above question is yes, to which of the following authority will you advise her to complain?	a. Treating doctor-24% b. Head of the institution/hospital-52.4% c. Police-5.1% d. Lawyer-1.2% e. Did not understand-17.4%
7.	When you visit a doctor with some problem do you always want to know about your diagnosis?	a. Yes-81.7% b. No -9.9% c. Did not understand -8.4%
8.	Can a person have access to her medical records file made at the time of her admission to the hospital/nursing home?	a. Yes- 58.7% b. No- 20% C. Did not understand - 21.3%
9.	Is there any time limit within which you need to file the case if you feel any medical malpractice?	a. Yes - 25.4% b. No - 32.6% c. Did not understand - 42%
10.	Do you think that all medical information that you share with your doctor is confidential and cannot be shared with anybody without your permission?	a. Yes - 74.3% b. No - 13.2% c. Did not understand - 12.6%
11.	Have you ever felt that lack of communication between you and your doctor is a reason for medical negligence?	a. Yes - 41.9% b. No - 39.2% c. Did not understand - 18.9%
12.	Do you think that informed written consent should be taken before any procedure?	a. Yes - 52.7% b. No - 17.4% c. Did not understand – 29.9%
13.	Can a general surgeon perform operations on the uterus or ovaries of women?	a. Yes - 11.7% b. No - 64.7% c. Did not know – 23.7%
14.	Do you think that the determination of the sex of the unborn child is appropriate?	a. Yes - 44.3% b. No - 43.4% c. Did not understand - 12.3%
15.	Are you aware of the Prevention of Preconception and Prenatal Determination of sex act?	a. Yes - 47.6% b. No - 28.4% c. Did not understand - 24%
16.	Will u like to know the sex of the unborn child in utero even if not allowed by the law?	a. Yes - 11.7% b. No – 75.4% c. Did not understand-12.9%
17.	If the answer to the above question is yes, why do you want to know the sex of the unborn child, Is it because	a. Just curiosity – 22.2% b. Preference – 14.4% c. Family pressure -3.6% d. Traditional beliefs -2.1% e. Did not answer – 57.8%
18.	Do you know that the doctors performing abortion has to be certified under the MTP Act?	a. Yes – 53.06% b. No – 14.07% c. Did not understand – 31.04%
19.	Do you know till what months/duration of pregnancy can it be aborted/ended lawfully?	a. Yes - 36.05% b. No – 37.07% c. Did not understand – 25.07%
20.	If yes, till what duration /months of pregnancy abortion/termination of pregnancy can be done lawfully?	a. 5 months – 29.06% b. 6 months - 13.02% c. 7 months - 13.02% d. 8months - 0% e. Did not answer – 57.2%
21.	Do you know till what month of pregnancy, one certified doctor can do termination of pregnancy (MTP)/abortion, and beyond that duration, two doctors are required to do MTP?	a. Yes – 26.3% b. No – 35.3% c. Did not understand -38.3%
22.	If yes, till what month of pregnancy, one certified doctor can do termination of pregnancy (MTP)/abortion, and beyond that duration, two doctors are required to do the MTPs?	a. 3months – 20.01 % b. 4months – 7.02% c. 5months – 3.9% d. 6months – 2.4% e. Did not understand – 66.5%
23.	Do you think that the written consent of the husband is required for family planning operation/sterilization?	a. Yes – 52.1% b. No – 28.04% c. Did not understand – 19.5%
24.	Legal age of marriage for boys?	a. 18 years - 1.8% b. 20 years - 2.1% c. 21 years - 70.07% d. 22 years - 22.08% e. Did not answer – 2.7%
25.	Legal age of marriage for girls?	a.18years-79.9% b.20years-7.5% c.21 years-9.3% d.22 years 0.6% e. Did not answer – 2.7%
